# Molecular evolution of Odorant-binding proteins gene family in two closely related *Anastrepha* fruit flies

**DOI:** 10.1186/s12862-016-0775-0

**Published:** 2016-10-07

**Authors:** Emeline Boni Campanini, Reinaldo Alves de Brito

**Affiliations:** Department of Genetics and Evolution, Federal University of São Carlos, Rodovia Washington Luís km235, 13565-905 São Carlos, São Paulo Brasil

**Keywords:** Sister species, Recent speciation event, Phylogenetic analyses, OBP subfamilies, OBP putative tertiary structure

## Abstract

**Background:**

Odorant-binding proteins (OBPs) are of great importance for survival and reproduction since they participate in initial steps of the olfactory signal transduction cascade, solubilizing and transporting chemical signals to the olfactory receptors. A comparative analysis of OBPs between closely related species may help explain how these genes evolve and are maintained under natural selection and how differences in these proteins can affect olfactory responses. We studied OBP genes in the closely related species *Anastrepha fraterculus* and *A. obliqua*, which have different host preferences, using data from RNA-seq cDNA libraries of head and reproductive tissues from male and female adults, aiming to understand the speciation process occurred between them.

**Results:**

We identified 23 different OBP sequences from *Anastrepha fraterculus* and 24 from *A. obliqua*, which correspond to 20 *Drosophila melanogaster* OBP genes. Phylogenetic analysis separated *Anastrepha* OBPs sequences in four branches that represent four subfamilies: classic, minus-C, plus-C and dimer. Both species showed five plus-C members, which is the biggest number found in tephritids until now. We found evidence of positive selection in four genes and at least one duplication event that preceded the speciation of these two species. Inferences on tertiary structures of putative proteins from these genes revealed that at least one positively selected change involves the binding cavity (the odorant binding region) in the plus-C *OBP50a*.

**Conclusions:**

*A. fraterculus* and *A. obliqua* have a bigger OBP repertoire than the other tephritids studied, though the total number of *Anastrepha* OBPs may be larger, since we studied only a limited number of tissues. The contrast of these closely related species reveals that there are several amino acid changes between the homologous genes, which might be related to their host preferences. The plus-C OBP that has one amino acid under positive selection located in the binding cavity may be under a selection pressure to recognize and bind a new odorant. The other positively selected sites found may be involved in important structural and functional changes, especially ones in which site-specific changes would radically change amino acid properties.

**Electronic supplementary material:**

The online version of this article (doi:10.1186/s12862-016-0775-0) contains supplementary material, which is available to authorized users.

## Background

The study of the genetics of species differences generally requires the identification of fixed genes between lineages. Most of these genes are simply “ordinary” traits that diverged between lineages with no direct role on isolation [1], while some could be involved with new changes or adaptations driven by selection. Up until recently, studies on speciation would then have to investigate these genes by interspecific crosses or other means to assess reproductive isolation [2] but new technical advances have enabled their identification by looking at selection at the gene level. Genes involved in reproduction and mate choice tend to evolve rapidly and display signatures of positive selection [3, 4], as well as genes facilitating chemoreception [5–8], because they are needed to interpret information about the environment, such as the presence of food or predators. Olfactory information, specifically, also controls social and sexual interactions between individuals of the same species, such as the detection of odors and pheromones essential for survival and reproduction [9]. In insects, the solubilization and transport of chemical signals through the aqueous lymph of insect’s sensilla to the olfactory receptors is the initial step of the transduction cascade of olfactory signals, and is mediated by the Odorant-binding proteins – OBPs [10].

OBPs are small soluble globular proteins (10–30 kDa), with six highly conserved cysteine residues (referred to as C1 to C6) in characteristic positions of all known insect OBPs. These cysteines form three disulphide bonds that stabilize their tertiary structure [11] and help define a hydrophobic binding cavity [12–14]. Different OBPs have specific affinities to odors and are present in distinctive portions of the sensilla, suggesting that they participate in odor detection by restricting the available spectrum of odors into adjacent receptors [11]. OBPs’ conserved cysteine residues, associated with functional information and phylogenetic relationships, have been used to classify OBPs into subfamilies: classic OBPs (six cysteines), plus-C OBPs (more than six cysteines) and minus-C OBPs (some members with less than six cysteines) [15–17]. Additionally, OBP transcripts encoding two complete OBP domains were identified in *Drosophila melanogaster*, and classified as a subfamily named dimer [17]. Other subfamilies were also defined in *Drosophila*: PBP, ABPI, ABPII, CRLBP and D7 that are unevenly distributed among dipterans [15, 18, 19]. It is expected that more generalist species would have a larger OBP repertoire to recognize various plant chemicals, in contrast to more specialist species, in which the power of natural selection to maintain a large OBP repertoire may have been weaker [20].

OBP genes are part of a gene family with low sequence similarity among its members, but high conservation at the structural level [21]. OBP genes are located in large gene clusters, suggesting that they arose by tandem duplication [22] and evolve mainly via the birth-and-death model [23], in which newly duplicated members progressively diverge in sequence and function, or may be lost to deletion or pseudogenization. The high disparity of gene sequences among OBPs implies a rapid rate of evolution in this gene family, suggesting that these genes might have evolved under the influence of positive selection [5, 8, 24], whereby even the conserved cysteine residues may be lost [17].

Here, we studied OBP genes in two closely related species of Tephritidae, *Anastrepha fraterculus* and *A. obliqua*, which are important fruit pests in South America. These species have diverged recently and exhibit a limited number of morphological [25] and genetic distinguishing characters [26]. Though *A. fraterculus* has been associated with a wide number of hosts, it prefers several Myrtaceae fruits [27], being considered one of the main economic pests in South America. *A. obliqua*, on the other hand, though an important pest species as well, has been associated to a smaller number of hosts, several of those Anacardiaceae [28]. Because OBPs are important targets for natural and sexual selection, their role in host and mate choice has previously been established in several species [29, 30]. The investigation of OBP evolution in these closely related species may provide clues about this group’s differentiation and host preference. We identified *A. fraterculus* and *A. obliqua* OBP members using RNA-seq data from reproductive and cephalic tissues of several different reproductive stages in male and female adults: before and after copulation and females after oviposition, and analyzed the patterns of molecular evolution between these two species. Several OBP genes were found to be evolving under positive selection and we speculate how these amino acid changes would affect protein structure and their consequences for adaptation.

## Methods

### Transcriptome libraries, assembly and annotation

We used populations of *A. fraterculus* from Southeast (22° 01′ 03″ S, 47 ^o^ 53′ 27″ W) and of *A. obliqua* from Midwest (16° 41′ 58″ S, 49 ^o^ 16′ 35″ W) regions of Brazil, that have been maintained in a controlled environment room at 25 °C ± 5 °C (60–90 % humidity) and natural photoperiod. Transcriptome libraries were generated for each species separately, using two reproductive stages for both sexes (virgin and post-mating), and a third one for females (post-oviposition). All profiles were made with biological replication, totaling 10 reproductive profiles per species. For each profile, we extracted the total RNA from head and reproductive tissues, totaling 20 libraries for each species. We used a pool of 10 individuals per library, amounting to 200 individuals in total.

More details on sample preparation, molecular procedures, assembly and annotation of the RNA-seq data are described elsewhere [31]. Briefly, total RNA was extracted using the TRizol/chloroform protocol [32]. RNA-seq libraries were constructed from four μg of total RNA using the TruSeq Stranded Total RNA Sample Prep Kit (Illumina) protocol, according to the manufacturer’s instructions. Pools of 12 libraries were run on an Illumina HiSeq2000 on a lane with runs of 2 × 100 bp paired-end reads. All reads were trimmed for quality and length with SeqyClean [33], keeping only reads with a minimum sequence length of 50 pb, a minimum of 0.01 for the parameter ‘max-avg-error’ and 0.05 for ‘max-error-at-ends’, and an average Phred quality score ≥ 20. Processed reads were assembled in two independent transcriptomes, one for each species, using the Trinity short read assembler (release 2013-02-25) [34], using default parameters.

We identified *A. fraterculus* and *A. obliqua* OBP sequences in the assemblies by BLASTx searches [35], using the Gene Ontology and *Drosophila melanogaster* database as a reference. Only the first match was considered, and contigs with an e-value threshold of 10^−5^ that corresponded to OBP genes were retained and their open reading frames inferred using ORF Finder [36]. We used PrediSi [37] to identify signal peptide sequences, since this is an important attribute of OBP proteins. OBPs of *A. fraterculus* and *A. obliqua* were designated as Afra and Aobl respectively, followed by the name of the respective OBP, based on their similarity with *D. melanogaster* OBPs (Table 1). Sequences associated with the same OBP were differentiated with a numerical postscript.

**Table 1:** Odorant-binding proteins identified in *A. fraterculus* and *A. obliqua* transcriptomes

*A. fraterculus* OBPs	ORF length (aa)	Signal Peptide (aa)	Accession Number	*A. obliqua* OBPs	ORF length (aa)	Signal Peptide (aa)	Accession Number
AfraOBP8a	156	1–20	KU317957	AoblOBP8a	156	1–20	KU317933
AfraOBP19a	149	1–26	KU317958	AoblOBP19a	149	1–26	KU317934
AfraOBP19b^NT^	130	NA	KU317976	AoblOBP19b	157	1–24	KU317935
AfraOBP19c	232	no	KU317959	AoblOBP19c	229	no	KU317936
AfraOBP19d	157	1–22	KU317960	AoblOBP19d	157	1–22	KU317937
AfraOBP47b	194	1–22	KU317961	AoblOBP47b	194	1–22	KU317938
AfraOBP49a	215	1–20	KU317962	AoblOBP49a-1	215	1–20	KU317939
				AoblOBP49a-2	178	1–19	KU317940
AfraOBP50a-1^NT^	139	NA	KU317977	AoblOBP50a	259	1–18	KU317941
AfraOBP50a-2^NT^	139	NA	KU317978				
AfraOBP50e	231	1–20	KU317963	AoblOBP50e	231	1–20	KU317942
AfraOBP56d-1^NT^	105	NA	KU317979	AoblOBP56d-1	138	1–18	KU317943
AfraOBP56d-2	138	1–18	KU317964	AoblOBP56d-2	138	1–18	KU317944
AfraOBP56h-1	136	1–19	KU317965	AoblOBP56h-1	136	1–19	KU317945
AfraOBP56h-2	125	1–19	KU317966	AoblOBP56h-2	125	1–19	KU317946
AfraOBP57c	178	1–50	KU317967	AoblOBP57c	178	1–50	KU317947
AfraOBP59a	309	1–26	KU317968	AoblOBP59a	309	1–26	KU317948
AfraOBP83cd	241	1–19	KU317969	AoblOBP83cd	241	1–19	KU317949
AfraOBP83ef	282	1–31	KU317970	AoblOBP83ef	282	1–31	KU317950
AfraOBP83g	143	1–17	KU317971	AoblOBP83g	143	1–17	KU317951
AfraOBP99a	143	1–17	KU317972	AoblOBP99a	143	1–17	KU317952
AfraOBP99b	153	1–17	KU317973	AoblOBP99b	153	1–17	KU317953
AfraOBP99c	151	1–17	KU317974	AoblOBP99c	151	1–17	KU317954
AfraOBP99d	152	1–20	KU317975	AoblOBP99d-1	151	1–20	KU317955
				AoblOBP99d-2	151	1–20	KU317956

^NT^N-terminus missing; *NA* not applied

### Alignments and phylogenetic analysis

We initially performed multiple alignment of the inferred OBP amino acid sequences for each species with MAFFT [38], using default settings. Average amino acid identities were obtained from the Percent Identity Matrix in the MAFFT alignment results. The multiple alignment of nucleotide sequences was manually adjusted in Bioedit v.7.0.5.3 [39], using the amino acid alignment as a guide. Three different methods, MaxChi [40], GENECONV [41] and RDP [42], were implemented in the RDP3 program [43] to investigate for recombination events.

Because OBPs are very divergent, we inferred the phylogenetic relationships in two steps. First, we aligned all *A. fraterculus* and *A. obliqua* OBPs here identified with OBP sequences of *Ceratitis capitata* and of *D. melanogaster* (obtained from GenBank - Additional file 1), using MAFFT as previously described. These species were chosen because *D. melanogaster* has the best curated genome and *C. capitata* is the closest species to *Anastrepha* with OBP sequences available. We used jModelTest [44] to estimate the best-fitting nucleotide model of substitution, that was used to infer Maximum likelihood phylogenetic relationships among OBPs with PhyML ver.3.0 [45]. This first phylogenetic tree was reconstructed in order to corroborate the annotation and, consequently, the allocation in subfamilies.

Based on the confirmation of the subfamilies’ division in our first phylogenetic tree, we performed a new alignment combining all *A. fraterculus* and *A. obliqua* OBPs and included OBP sequences from the tephritids *C. capitata*, *Bactrocera dorsalis* [46], *Rhagoletis pomonella* [47], and *Rhagoletis suavis* [48]*.* This alignment was performed with MAFFT separating and aligning OBP sequences by subfamily, in such a way that the alignments by subfamilies were combined in one general alignment using the Profile Alignment Mode in ClustalX2 [49], which produced a better alignment and a second phylogenetic tree, which was inferred as previously described. MAFFT also provided us with an identity matrix, which we used to estimate the average amino acid identity by subfamily. Due to the high divergence in the N-terminal region, for the phylogenetic and evolutionary analysis we removed the region before the first cysteine residue in all sequences prior to the alignments, as it was done in other studies [19, 50–52].

### Evolutionary analysis

Since OBPs from different subfamilies show great divergence, we chose to perform the evolutionary analysis by subfamilies. We used separate alignments and phylogenetic trees for each one of the four OBP subfamilies using sequences of *A. fraterculus*, *A. obliqua*, *C. capitata* and *D. melanogaster* (Additional file 2). Before phylogenetic reconstruction, we tested the alignments for sequence saturation using DAMBE 5 [53]. We investigated patterns of molecular evolution and positive selection with the strict branch-site test comparing models A vs*.* A-null [54], using the software CODEML, implemented in PAML v.4 [55]. The comparison between models was tested using likelihood-ratio tests (LRTs) for hierarchical models [56], and we used a Bonferroni correction for the number of branches tested. A significantly higher likelihood for the alternative model than that of the null model would indicate evidence of positive selection. We also estimated pairwise Ka, Ks rates and its ratio Ka/Ks, using the KaKs_Calculator [57], for the putative orthologous genes using the MS model [58]. Genes showing Ka/Ks rates higher than 0.5 were considered as potentially evolving under positive selection [59].

The Bayes Empirical Bayes (BEB) method implemented in PAML was used to estimate the posterior probability that a given site is evolving under positive selection. Furthermore, we analyzed whether amino acid replacements in positively selected sites alter physicochemical properties in the proteins using Conant-Stadler amino acid property set [60] with the PRIME method available in the Datamonkey web server [61]. To make inferences about the position of the BEB positively selected sites on the tertiary structure of the proteins, we predicted the tertiary structure of *Anastrepha* OBPs under positive selection with PHYRE2 [62]. The program used *Ae. aegypti* OBP1 (PDB: 3K1E, chain A [12]) as reference to infer the tertiary structure of *Anastrepha OBP56h-1* and *OBP56h-2*, and *A. gambiae* OBP4 (PDB: 3Q8I, chain A [63]) and *A. gambiae* OBP48 (PDB: 4KYN, chain B [14]) as reference to *OBP57c* and *OBP50a*, respectively. Since gene sequences in both species were similar and because *AfraOBP50a-1* and *AfraOBP50a-2* were incomplete, we used *A. obliqua* sequences to make the predictions.

## Results and discussion

### OBP genes in *Anastrepha fraterculus* and *Anastrepha obliqua*

We identified a similar number of sequences associated with OBPs in the two species: 23 different sequences in *A. fraterculus* and 24 in *A. obliqua*, which corresponded to 20 different *D. melanogaster* OBP genes (Table 1). In comparison, free-living insect OBP families described to date range from 11 genes in the beet armyworm *Spodoptera exigua* [64] to more than a hundred in some species of mosquitoes [65], although the parasitic body louse *Pediculus humanus* (which has a reduced genome), has only five OBPs [66]. The length of complete open reading frames (Table 1) matched what has been described for several arthropod species [67]. The longest OBPs are *AfraOBP59a* and *AoblOBP59a*. These sequences are unusual, because they have a specific region containing 134 amino acids that is situated between the first (C1) and the second (C2) cysteine, whereas others in the same subfamily have ~30 amino acids in this region. Even though an unusual length for *OBP59a* had already been demonstrated in other arthropods [67], in *Anastrepha* these OBPs are 106 amino acids longer than in *D. melanogaster*. Signal peptides, one of characteristic hallmarks of the OBP gene family, were predicted at the hydrophobic N-terminal for the almost all OBPs, except for *AfraOBP19c* and *AoblOBP19c*. Though not all OBPs described have signal peptides [52], we believe that the PrediSi program failed to find a match in the SwissProt database for both *Anastrepha OBP19c* signal peptide due to sequence divergence, not because these OBPs lack a signal peptide.

A comparison of putatively homologous OBPs in *A. fraterculus* and *A. obliqua* reveals great amino acid similarity, so much so that homologs of *OBP19a* and *OBP99a* had identical amino acid sequences in both species. In spite of that, the majority of orthologous OBPs differ by at least a few amino acids, similar to what was described for related species of aphids [52, 68]. However, even a single amino acid change may impact the overall 3D structure and/or the binding affinities of OBPs [69, 70]. For instance, a polymorphism in *Obp57e* was shown to be responsible for differences in host plant preferences between *D. sechellia* and *D. melanogaster* [29], and some polymorphisms were associated with natural variation in olfactory behavior in response to benzaldehyde in *Obp99a*, *Obp99c*, and *Obp99d* in *D. melanogaster* [71]. Likewise, the few changes observed between *A. fraterculus* and *A. obliqua* OBPs may result in significant differences in olfactory responses, which is yet to be determined.

We did an alignment with all *A. fraterculus* OBP sequences, and another one with all *A. obliqua* OBP sequences. Two independent recombination tests were performed using these alignments, because recombination interferes with phylogenetic inferences and may generate higher rates of false positives in positive selection tests [72]. Both tests performed failed to find significant evidence for recombination. These alignments revealed that even though putatively orthologous OBP copies in different species were very similar, there was great divergence among OBP genes, so much so that only the cysteine residues were conserved across all OBPs of the same species. Sequence divergence was higher than what has been described for other insects. The average amino acid identity among all OBPs was 16.65 % in *A. fraterculus* (ranging from 8.1 to 97.1 %) and 16.22 % in *A. obliqua* (varying from 8.0 to 98.7 %), compared with 20.4 % of identity between all OBPs in *D. melanogaster* [15], and 20 % between all OBPs found in *Solenopsis invicta* [73].

The higher intraspecific divergence among OBPs in *A. fraterculus* and *A. obliqua*, when compared with other insects, may suggest a greater selective pressure for OBP differentiation, or relaxed selection, which also led us to investigate for patterns of evolution in these genes. On the other hand, the high amino acid similarity found between the homologs in *A. fraterculus* and *A. obliqua* probably indicates that the recent time of divergence between these species may not have been enough to drastically alter their sequences, which could be a reflection of retention of similar physiological functions across species or simply of the recent time since their divergence. Since these species have diverged recently and have accumulated only a few changes, the current methodologies to investigate for positive selection between these species are not very efficient, but we may use these data in contrast to other Tephritidae and Drosophilidae to investigate for patterns of selection in OBPs as a whole. We chose to investigate *Anastrepha* OBPs because they may be involved with the group’s differentiation since they may be directly involved with host preference and mate choice. Therefore, patterns of selection in these genes may help us understand evolution and speciation not only in the genus *Anastrepha*, but also across this important family, which may make it useful in pest control programs.

### Phylogenetic relationships among OBP genes

**Fig. 1:**
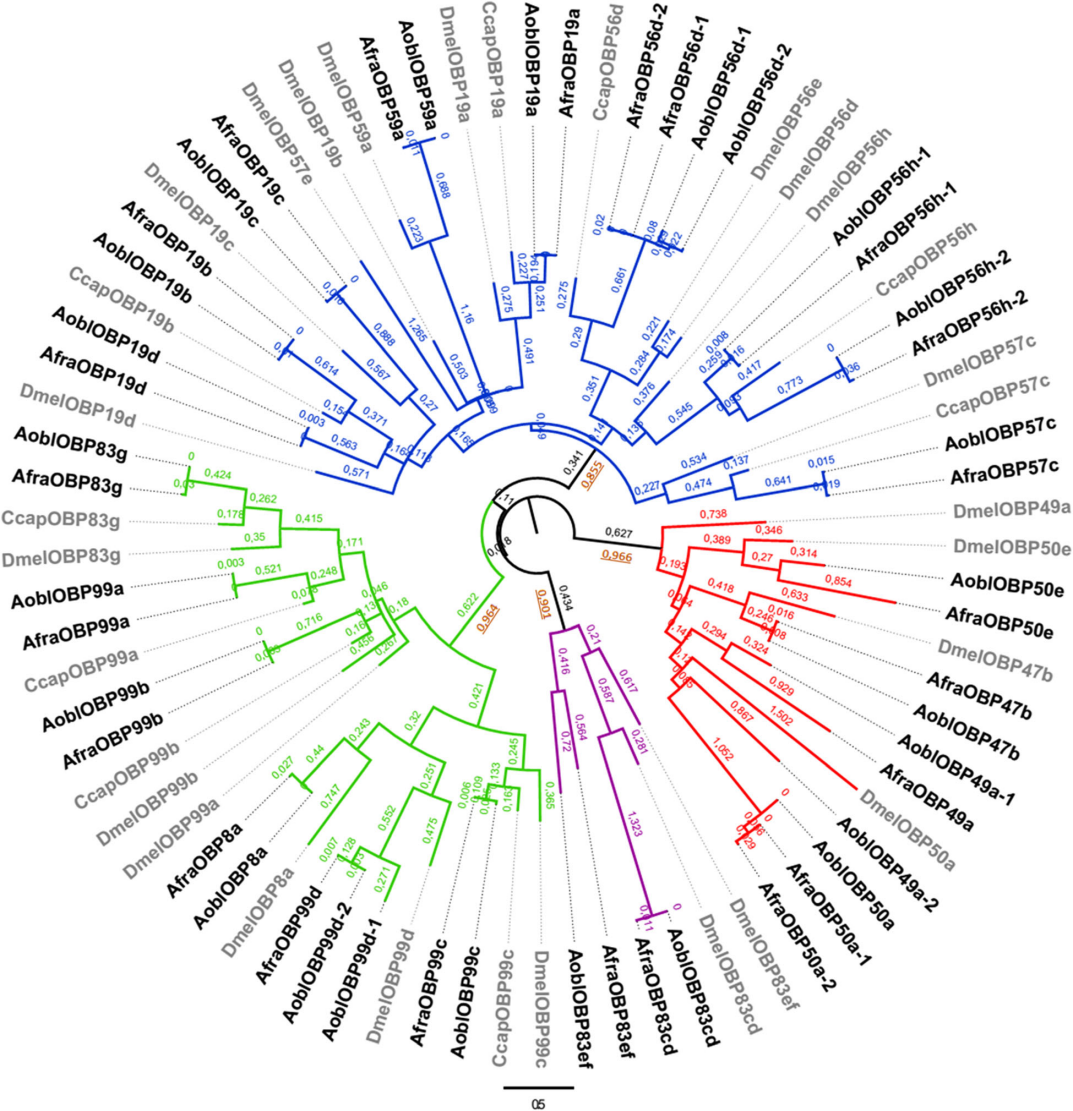
Mid-point rooted ML phylogenetic relationships of the OBPs. The branches are color coded for each subfamily: classic (*blue*), minus-C (*green*), plus-C (*red*) and dimer (*purple*). Branch lengths are estimated by amino acid substitutions per site. *Brown* and *underlined numbers* represent the SHlike aLRT branch supports for the four subfamilies

A ML phylogenetic tree that included all OBP sequences derived from *Anastrepha, C. capitata* and *D. melanogaster* (Fig. 1) indicates four clades that correspond to four distinct protein subfamilies based on *D. melanogaster*’s classification [15], hence the importance of including this species in the analysis. The four clades were highly supported (SHlike aLRT branch supports ranging from 0.855 and 0.966) and were composed of OBPs from all species here investigated, indicating that these subfamilies diverged before their common ancestor, which has already been described for others dipterans [19, 20, 74]. The support for each separate OBP subfamily in *Anastrepha* was also confirmed by the conservation of cysteine patterns (Table 2).

**Table 2:** Attributes of *Anastrepha* Odorant-binding proteins subfamilies

	Subfamily	Number of members	ORF length (aa)	Protein core region	Amino acid identity (%)
*A. fraterculus*	classic	10	125–309	C1 - X_24–33_ - C2 - X_3_ - C3 - X_35–47_ - C4 - X_8–18_ - C5 - X_8_ - C6	28.20
	minus-C	6	143–156	C1 - X_26_ - C2 - X_3_ - C3 - X_39–40_ - C4 - X_10_ - C5 - X_8_ - C6 and C1 - X_23–30_ - C3 - X_38_ - C4 - X_18–19_ - C6	39.14
	plus-C	5	194–231	C1a - X_11–13_ - C1b - C1c - C1 - X_21–48_ - C2 - X_3_ - C3 - X_43_ - C4 - X_20–33_ - C5 - X_9_ - C6 - X_8_ - C6a - X_10_ - C6b - X_9_ - C6c	45.14
	dimer	2	241–282	C1 - X2_8–29_ - C2 - X_3_ - C3 - X_31_ - C4 - X_10–11_ - C5 - X_8_ - C6 - X_17–28_ - C1′ - X_24–25_ - C2′ - X_3_ - C3′ - X_35_ - C4′ - X_9–12_ - C5′ - X_8_ - C6′	65.75
*A. obliqua*	classic	10	125–309	C1 - X_24–37_ - C2 - X_3_ - C3 - X_35–42_ - C4 - X_8–12_ - C5 - X_8_ - C6	29.54
	minus-C	7	143–156	C1 - X_26_ - C2 - X_3_ - C3 - X_39–40_ - C4 - X_10_ - C5 - X_8_ - C6 and C1 - X_23–30_ - C3 - X_38_ - C4 - X_18_ - C6	38.10
	plus-C	5	178–259	C1a - X_11–13_ - C1b - C1c - C1 - X_21–48_ - C2 - X_3_ - C3 - X_43_ - C4 - X_20–33_ - C5 - X_9_ - C6 - X_8_ - C6a - X_10_ - C6b - X_9_ - C6c	40.25
	dimer	2	241–282	C1 - X_28–29_ - C2 - X_3_ - C3 - X_31_ - C4 - X_10–11_ - C5 - X_8_ - C6 - X_17–28_ - C1′ - X_24–25_ - C2′ - X_3_ - C3′ - X_35_ - C4′ - X_9–12_ - C5′ - X_8_ - C6′	65.14

Both protein core regions are reported for the minus-C subfamily, with four and six conserved cysteine residues

The average amino acid identity was higher in the dimer subfamily for both species, but the number of members in each subfamily may have inflated these values, since larger families show lower average identity (Table 2). Classic OBPs have the expected standard pattern of six conserved cysteine residues, whereas dimer OBPs have twelve cysteine residues, a pattern formed by the junction of two consecutive minus-C OBP domains [8]. The dimer subfamily was identified in *A. fraterculus*, *A. obliqua* and *D. melanogaster*, though it has not been described for *C. capitata* yet. As in *D. melanogaster*, *A. fraterculus* and *A. obliqua* minus-C OBPs were divided in two lineages, one in which the members retain six conserved cysteine residues (a clade that includes *OBP8a*, *OBP99c* and *OBP99d*), and another one (that includes *OBP83g*, *OBP99a* and *OBP99b*) in which its members show only four conserved cysteine residues (Fig. 1). The reduced number of residues in some members is caused by the loss of the conserved pair of cysteines C2 and C5, which forms a disulphide bond [75]. That happened after the minus subfamily diverged from the classic subfamily and might have a functional relevance, perhaps generating a more flexible structure [15, 19].

*A. fraterculus* and *A. obliqua* plus-C OBPs showed three additional conserved cysteine residues before C1 (referred to as C1a, C1b and C1c) and three others after C6 (C6a, C6b and C6c), making for a total of 12 conserved cysteine residues. Three sequences associated with *OBP50a* did not have the cysteine C6c. Similar to what was described for *Drosophila*, we found a conserved hydrophobic proline after the cysteine C6a in *Anastrepha*’s plus-C members, as well as nine residues between C5 and C6, instead of eight in the other subfamilies [19]. Recently, Siciliano et al. [74] reported a plus-C OBP in *C. capitata*, related to *DmelOBP49a*, but in their phylogenetic tree, this OBP was grouped with classic OBPs. OBPs in the plus-C subfamily, such as the ones described here, generally show a substantial increase in length size compared with other OBPs, mostly because of the extended C-termini [5, 16]. The plus-C subfamily was also described in the pea aphid *Acyrthosiphon pisum* [52], and the high support in the tree indicates that their origin probably precedes the divergence of Diptera.

**Fig. 2:**
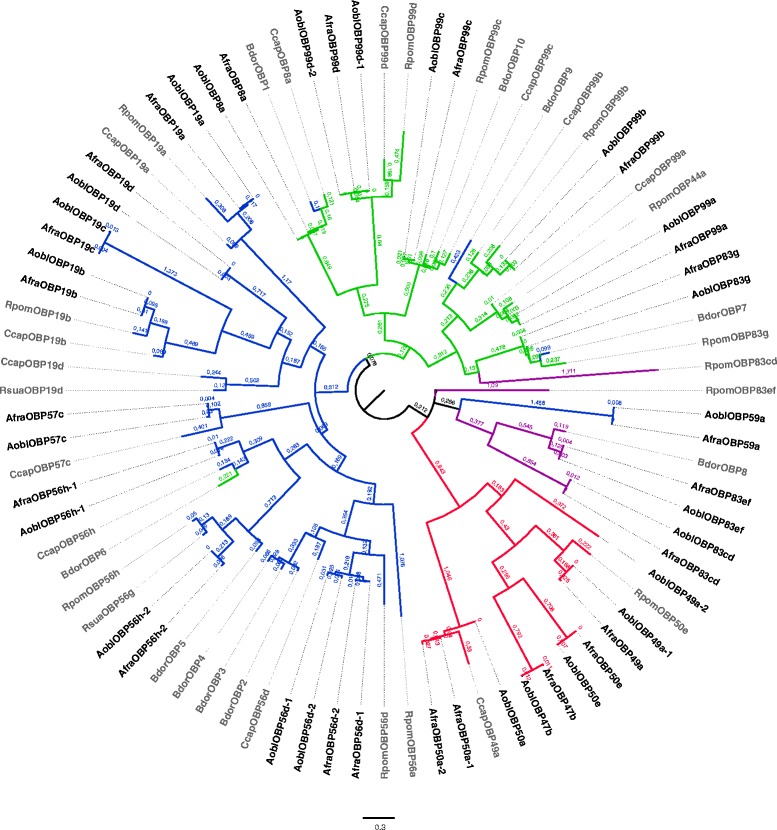
Mid-point rooted ML phylogenetic relationships of the tephritids OBPs. The branches are color coded for each subfamily: classic (*blue*), minus-C (*green*), plus-C (*red*) and dimer (*purple*). Branch lengths are estimated by amino acid substitutions per site

We also performed a phylogenetic inference that was restricted to tephritid OBPs, including *A. fraterculus*, *A. obliqua*, *C. capitata*, *B. dorsalis*, *R. pomonella* and *R. suavis* (Fig. 2) which would enable us to have a better understanding of the patterns of evolution of OBPs in these important pest species. As in the previous phylogenetic inference (Fig. 1), we observed a basal division in four subfamilies with one exception. *Anastrepha OBP59a*, a classic OBP, in this inference was grouped with the dimer subfamily members, probably due to its extended length, which could have led to long-branch attraction. We also observed that *RpomOBP83cd*, a dimer member, grouped with minus-C subfamily, and some *B. dorsalis* OBPs were grouped in different subfamilies than was previously described [46]. In our phylogenetic inference of tephritid OBPs, *CcapOBP49a* was grouped with plus-C subfamily members (SHlike aLRT branch support 0.992 and branch length 1.046), not with the classic subfamily, as previously described [74]. In general, each *Anastrepha* OBP clustered with their putative homolog from other tephritid species, with high support for the majority of branches.

Even though the overall number of OBP genes seems to be equivalent among tephritids, we point out that species of *Anastrepha* have a larger OBPs repertoire than the other tephritids studied: 23 and 24 OBP genes, respectively. In comparison, 17 different OBPs were described for *C. capitata,* the closest species of *A. fraterculus* and *A. obliqua* studied, 13 of which corresponded to the classic OBP subfamily, three to the minus-C subfamily and one to the plus-C subfamily [74]. We also found a larger number of plus-C OBPs when compared to other tephritids. We detected five apiece for *A. fraterculus* and *A. obliqua*, whereas two were described in *R. pomonella* and only one in *R. suavis*, *B. dorsalis* and *C. capitata*. The lack of genomic sequences for *Anastrepha* makes the analyses here not complete, since we may only rely on levels of divergence and phylogenetic expectations that could be verified solely upon investigation of the evolutionary patterns found in the species’ genome. It is possible that upon the availability of genome sequences, we may detect a wider range and number of OBP genes. 

We observed some instances in which more than one sequence in the *A. fraterculus* and *A. obliqua* OBP repertoire was associated with the same *D. melanogaster* OBP. In *A. fraterculus*, two different sequences were associated with the genes *OBP50a*, *OBP56d* and *OBP56h*, and in *A. obliqua*, with the genes *OBP49a*, *OBP56d*, *OBP56h* and *OBP99d*. These findings could be due to gene duplication events, though we cannot rule out intraspecific variation, because we used a pool of flies from a single population to make the cDNA libraries in each species. Analyzing the values of pairwise identity between the two copies of the same OBP of a species and comparing copies of the same OBP between species, different *Anastrepha* sequences associated with the same OBP seem to be due to intraspecific variation, with the exception of two cases: *OBP56h* and *OBP49a*.

We believe that the copies homologous to *OBP56h* are consequence of a duplication event that preceded the divergence of *A. fraterculus* and *A. obliqua. AfraOBP56h-1* and *AoblOBP56h-1* have a pairwise identity of 93.6 %, and *AfraOBP56h-2* and *AoblOBP56h-2* an identity of 96.3 %, whereas the pairwise identity between *OBP56h* sequences of the same species is 29.3 % in *A. fraterculus* and 33.3 % in *A. obliqua*, clearly indicating them to be paralogous. Moreover, the phylogenetic analysis of plus-C members reveals that *AoblOBP49a-2* groups with the other *OBP49a*, including *DmelOBP49a* (Additional file 2). However, *AoblOBP49a-2* is 37 amino acids shorter than *AoblOBP49a-1* and *AfraOBP49a* and quite different. While the pairwise amino acid identity between *AoblOBP49a-1* and *AfraOBP49a* is 95 %, it is only 31 % between the two copies of *A. obliqua* (*AoblOBP49a-1* and *AoblOBP49a-2*), suggesting as well that *AoblOBP49a-2* is not an ortholog of *DmelOBP49a*, such as *AoblOBP49a-1* and *AfraOBP49a* but, rather, a paralog. The fact that we failed to find this copy in *A. fraterculus* may indicate that it diverged after the separation of the species, which would be extremely interesting, but considering the high levels of divergence found among these paralogs, it is more likely that they diverged before the separation of *Anastrepha* species, and did not show significant levels of expression in *A. fraterculus*, which is also relevant.

### Analysis of positive selection in OBPs

Similar to all insect OBPs studied so far [73], *Anastrepha* OBPs share low sequence similarity, rendering evolutionary analyses difficult. Therefore, we did not use the alignment that included all subfamilies because we identified that there was saturation at synonymous positions in more divergent comparisons (data not shown), which might lead to a higher number of false positives in the branch-site test of positive selection [76]. We performed independent evolutionary analyses on each of the four OBP subfamily identified (Table 2; Additional file 2), which did not show evidence of sequence saturation. This, unfortunately, prevented us from investigating evidence of positive selection amongst the four subfamilies, but allowed us to investigate for positive selection within any of such subfamily.

We detected evidence of positive selection, using the strict branch-site test, for sequences associated to two classic OBP genes: *OBP56h* (*AfraOBP56h-1* and *AoblOBP56h-1* separated from *AfraOBP56h-2* and *AoblOBP56h-2* on different branches) and *OBP57c* (*AfraOBP57c* and *AoblOBP57c*). The significant results in the strict branch-site test for *Anastrepha OBP56h* suggest that after the likely duplication event in this gene, indicated by the pairwise identity analysis, positive selection acted to differentiate the two copies, which could have been to discrete odor specificities. Moreover, we detected positive selection on one plus-C gene: *OBP50a* (*AfraOBP50a-1*, *AfraOBP50a-2* and *AoblOBP50a*) (Table 3). The *OBP50a* copies from *A. fraterculus* are more closely related to one another than to the corresponding copy from *A. obliqua*, and have over 97 % similarity, which could be an indication of intraspecific polymorphism in *A. fraterculus*. However, the evidence of positive selection acting in these genes may indicate a recent duplication in that species, which could be best investigated by an analysis on the genome. Contrary to other insects such as *Apis mellifera* and *B. mori* [5, 51], we failed to find evidence of positive selection on the minus-C subfamily.

**Table 3:** Positive selection detected in *Anastrepha* OBP genes

Gene	LRT^a^	Positively selected sites^b^
*AfraOBP56h-1* *AoblOBP56h-1*	10,444**	85
*AfraOBP56h-2* *AoblOBP56h-2*	7555**	40, 67, 68, 145, 152
*AfraOBP57c* *AoblOBP57c*	8403**	5, 34, 61, 65, 76, 94, 146, 150
*AfraOBP50a-1* *AfraOBP50a-2* *AoblOBP50a*	8416*	4, 56, 59, 146, 152, 156, 164, 177, 181

^a^Likelihood Ratio Test results; ^b^These numbers reflect the site positions in the overall alignment, not necessarily the position in each specific OBP. Amino acid positions involving changes in the physicochemical properties influenced by positive selection are underlined. **p < 0.00625; *p < 0.0125

**Fig. 3:**
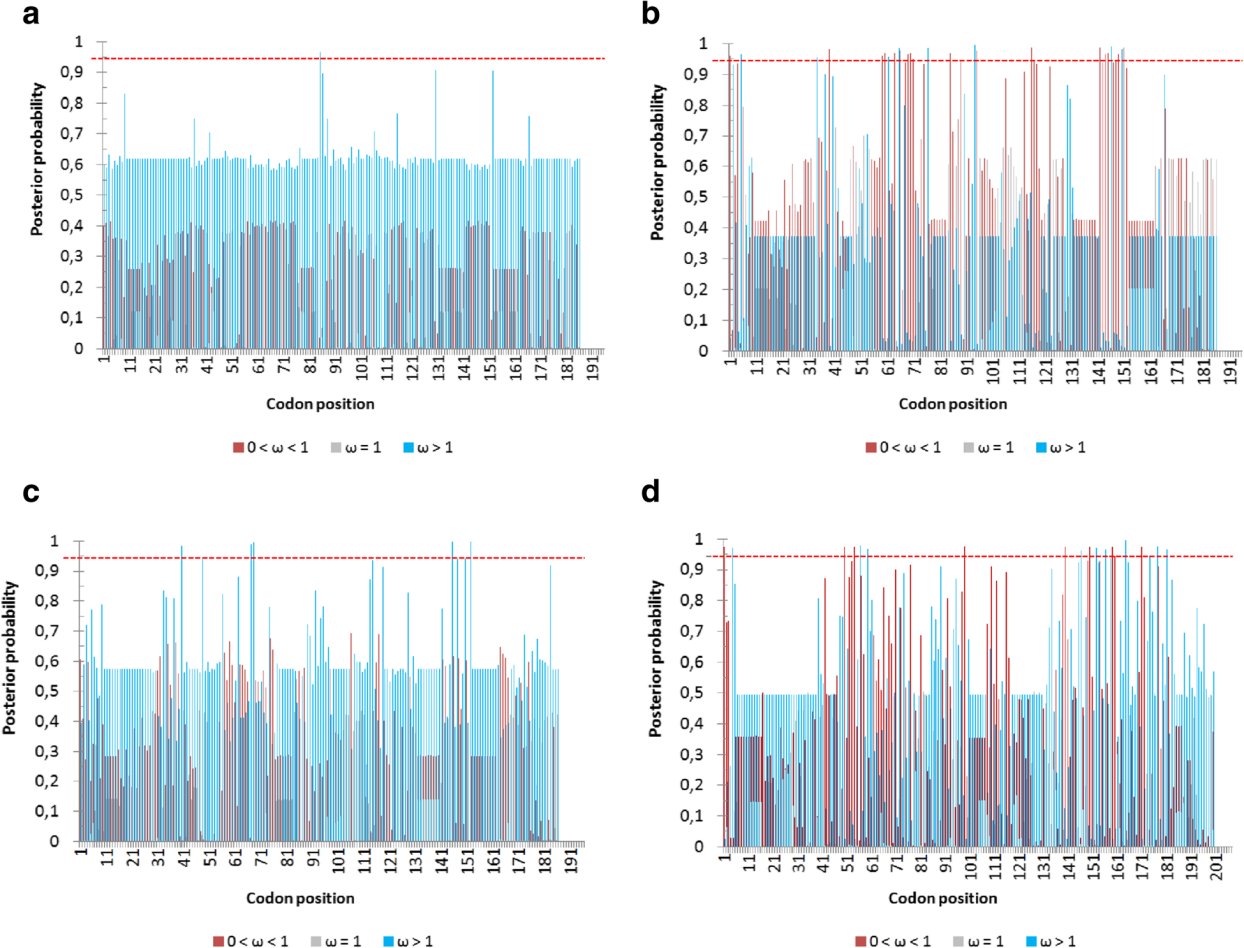
Bayes Empirical Bayes showing posterior probabilities of sites under purifying (0 < ω < 1), neutral (ω = 1) and positive selection (ω > 1). Amino acid in codon position ‘1’ is cysteine C1. *Dashed red lines* indicate the posterior probability threshold of 0.95. **a**) results for *AoblOBP56h-1* and *AfraOBP56h-1*; **b**) results for *AoblOBP56h-2* and *AfraOBP56h-2*; **c**) results for *AoblOBP57c* and *AfraOBP57c*; **d**) results for *AoblOBP50a*, *AfraOBP50a-1* and *AfraOBP50a-2*

A Bayes Empirical Bayes (BEB) method identified several amino acid sites under positive selection (Fig. 3; Table 3), some of which were in regions under strong purifying selection (Fig. 3b; d). For classic OBP sequences associated with *OBP56h* and *OBP57c*, the positively selected amino acids were located in the protein core region (between cysteines C1 and C6), except for the amino acid in position 152, the position right after C6. For plus-C *OBP50a,* we found the largest number of positively selected sites: nine sites, four of them located in the protein core region. The amino acid in position 181 is the cysteine C6c (the twelfth cysteine residue) in all plus-C sequences except in those associated with *OBP50a*, where a cysteine was replaced by asparagine. Though in general cysteines are conserved in OBPs, even highly conserved cysteine residues may be lost [17].

We investigated whether amino acid changes in OBPs detected by the BEB analysis were associated with site-specific changes related to five amino acid properties: chemical composition, polarity, volume, isoeletric point and hydropathy (*p* < 0.05), because not all non-synonymous substitutions are alike. If non-synonymous substitutions result in similar properties, the maintenance of the structure and/or chemical function of the region bearing such changes would be considered as conserved. On the other hand, radical non-synonymous changes drastically change important physicochemical attributes. When these changes involve sites under positive selection, it is more likely that they would be those that promote structural and functional changes in a protein. Among the five non-synonymous changes in the duplicated copy of *Anastrepha OBP56h-2* we found two amino acid positions (68 and 145) that are associated with radical changes in the physicochemical properties, one associated to changes in isoelectric point, and another to changes in chemical composition. The high number of amino acid substitutions, particularly leading to radical changes, may be an indication of the effect of positive selection driving this recently duplicated copy to a different odor affinity.

*Anastrepha OBP57c* and *Anastrepha OBP50a* also have one amino acid position each associated with radical changes in the physicochemical properties. A change in the amino acid position 150 of *Anastrepha OBP57c* alters its isoeletric point, whereas a change in amino acid 59 of *Anastrepha OBP50a* affects its volume. Changes in chemical composition and volume of OBPs are important because they may be related to conformational changes in these globular proteins. Altering the structure of α-helices may modify their flexibility and, therefore, modify the interacting motifs of the protein [8]. Likewise, substitutions that affect the isoeletric point are important since they may be involved with changes in the proteins’ solubility, which, in the aqueous lymph of sensillas, may be related with a greater efficiency in transport activity.

An analysis of synonymous and non-synonymous substitution rates between putatively homologous copies in *A. fraterculus* and *A. obliqua* reveals several copies with high values for Ks (Table 4) indicating that even though the number of changes between homologous copies may not be high, there is a high proportion of non-synonymous changes. We have identified two OBPs with Ka/Ks above one and seven others that had Ka/Ks over 0.5, whereas the median Ka/Ks ratio in putatively orthologous OBPs is 0.44. Though we should consider that the Ka/Ks ratio may not be reliable when mutation numbers are small, e.g., for within populations estimates [77] or even for recently diverged lineages, which seems to be the case here, a comparison of these data with other genes in the same populations may provide an interesting parameter to consider when evaluating neutral evolution. A similar analysis performed on 164 contigs that were identified as highly differentiated between *A. fraterculus* and *A. obliqua* shows median Ka = 0.0026, Ks = 0.0282 and Ka/Ks = 0.09 and 11 contigs with a Ka/Ks above 0.5, one of them an OBP [31]. A contrast between these two data sets reveals that OBPs as a whole evolve faster and have proportionately more non-synonymous substitutions between these two species than other genes in the genome (*t*-test = 3.74, *p* < 0.001) and suggests that several OBPs may be evolving under positive selection, though we cannot rule out that they are evolving under relaxed selection.

**Table 4:** Estimates for Ka, Ks and Ka/Ks ratio between *A. fraterculus* and *A. obliqua* putatively orthologous OBP genes

OBP genes compared	Ka	Ks	Pairwise Ka/Ks	*P*-value (Fisher)
*AfraOBP8a - AoblOBP8a*	0.01302	0.04044	0.32192	0.02418
*AfraOBP19a - AoblOBP19a*	0.00001	0.01018	0.00100	0.08109
*AfraOBP19b - AoblOBP19b*	0.01250	0.02512	0.49751	0.34228
*AfraOBP19c - AoblOBP19c*	0.02404	0.01055	2.27963	0.35887
*AfraOBP19d - AoblOBP19d*	0.00275	0.00005	50	0.36788
*AfraOBP47b - AoblOBP47b*	0.01987	0.05524	0.35971	0.03121
*AfraOBP49a - AoblOBP49a-1*	0.01922	0.04152	0.46284	0.04973
*AfraOBP49a - AoblOBP49a-2*	0.75439	1.89710	0.39765	0
*AfraOBP50a-1 - AoblOBP50a*	0.03385	0.12629	0.26803	0.00095
*AfraOBP50a-2 - AoblOBP50a*	0.04541	0.22418	0.20258	0
*AfraOBP50e - AoblOBP50e*	0.00448	0.03749	0.11959	0.00282
*AfraOBP56d-1 - AoblOBP56d-1*	0.06552	0.15216	0.43063	0.0365
*AfraOBP56d-2 - AoblOBP56d-2*	0.14590	0.20809	0.70117	0.13362
*AfraOBP56d-1 - AoblOBP56d-2*	0.06276	0.12104	0.51850	0.12677
*AfraOBP56d-2 - AoblOBP56d-1*	0.14717	0.21836	0.67400	0.10769
*AfraOBP56h-1 - AoblOBP56h-1*	0.01884	0.05216	0.36124	0.07521
*AfraOBP56h-2 - AoblOBP56h-2*	0.03487	0.04064	0.85809	0.72667
*AfraOBP56h-1 - AoblOBP56h-2*	0.70700	1.99231	0.35522	0
*AfraOBP56h-2 - AoblOBP56h-1*	0.72197	1.84218	0.39191	0
*AfraOBP57c - AoblOBP57c*	0.02325	0.03906	0.59518	0.36258
*AfraOBP59a - AoblOBP59a*	0.00409	0.02980	0.13711	0.00594
*AfraOBP83cd - AoblOBP83cd*	0.00951	0.02519	0.37748	0.16455
*AfraOBP83ef - AoblOBP83ef*	0.00202	0.01555	0.13002	0.04322
*AfraOBP83g - AoblOBP83g*	0.02066	0.06384	0.32355	0.08097
*AfraOBP99a - AoblOBP99a*	0.00002	0.02374	0.00100	0.01413
*AfraOBP99b - AoblOBP99b*	0.00826	0.01162	0.71080	0.33160
*AfraOBP99c - AoblOBP99c*	0.02607	0.05666	0.46011	0.06971
*AfraOBP99d - AoblOBP99d-1*	0.01599	0.02393	0.66817	0.40804
*AfraOBP99d - AoblOBP99d-2*	0.01000	0.02179	0.45910	0.12015
Median	0.01902	0.03975	0.44486	

### Positively selected sites in OBP 3-D structures

Positively selected sites experience a faster rate of amino acid replacement that is a consequence of mutations being fixed at a higher rate than expected by chance. Therefore, knowing the location of these amino acid sites in the protein’s tertiary structure may help understand how these changes would affect their function. Structurally, OBPs share a common fold with α-helices connected by loops and interlinked by disulphide bonds, but despite their structural homology, they are predicted to bear binding cavities of different shapes [21]. Positive selection in OBPs may promote functional divergence on the binding specificities, which is important because such structural diversity may enable OBPs to recognize and bind to a wider range of organic molecules and naturally occurring odorants [8].

**Fig. 4:**
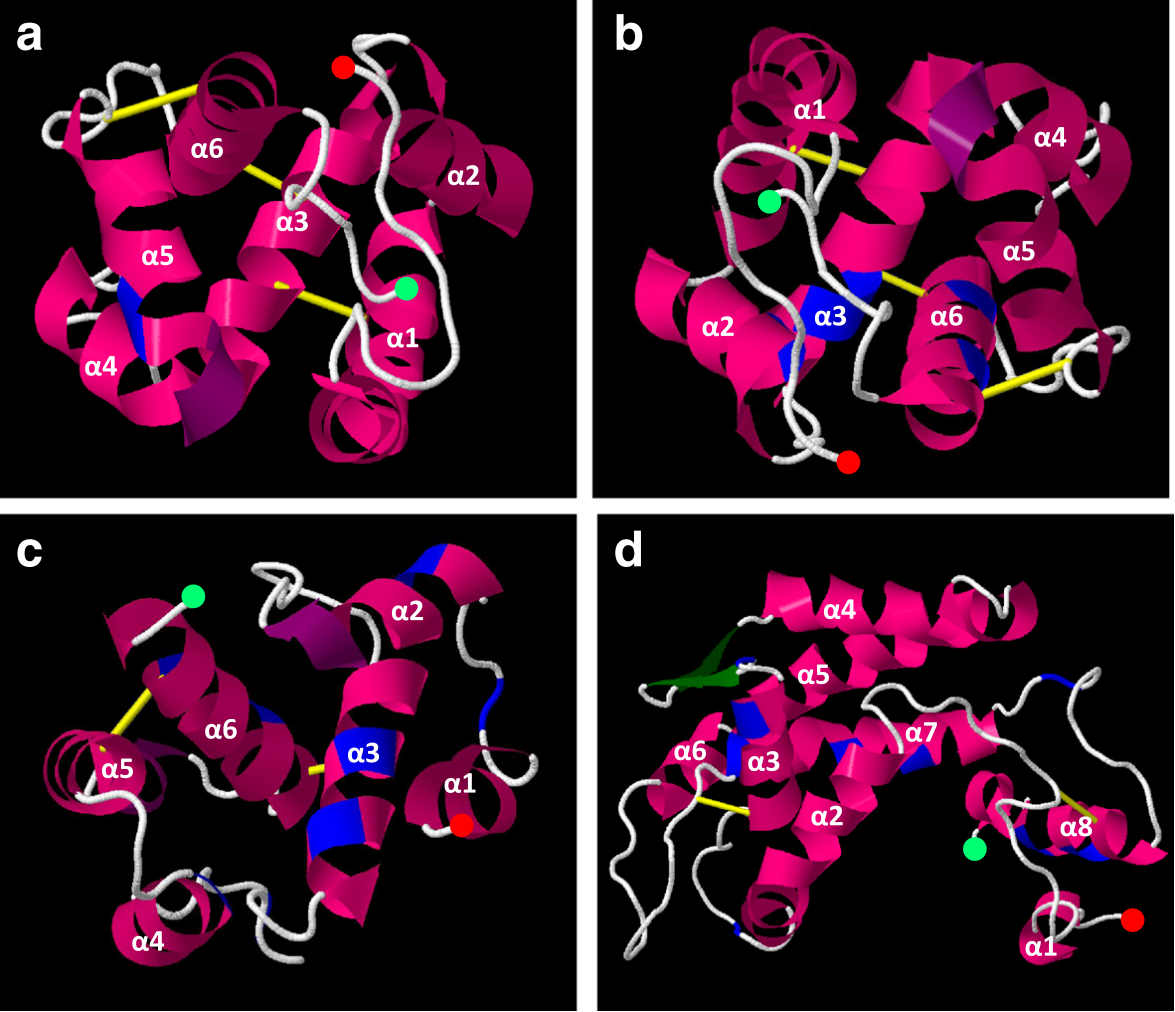
Cartoon representation of hypothetical positions of the positively selected sites in *Anastrepha* OBPs, based in their predicted 3D structures. α-helices are shown in *pink*, β-sheets in *green*, loops in *white*, disulphide bonds in *yellow* and positively selected sites in *blue*. N- and C-terminus residues are shown with *red* and *light green circles*, respectively. **a**) *AoblOBP56h-1* and *AfraOBP56h-1* representation; **b**) *AoblOBP56h-2* and *AfraOBP56h-2* representation; **c**) *AoblOBP57c* and *AfraOBP57c* representation; **d**) *AoblOBP50a*, *AfraOBP50a-1* and *AfraOBP50a-2* representation

The predicted 3D structures were used to locate the amino acid positions under positive selection according to PAML analysis (Fig. 4). The tertiary structure of *Ae. aegypti OBP1*, selected by Phyre2 as reference for *Anastrepha OBP56h* due to their similarity, is a protein with 125 amino acid residues, comprised of six α-helices connected by loops between helices and knitted together by three disulphide bridges between α1 and α3, α3 and the top of α6, and α6 and the top of α5. Sixteen hydrophobic residues in helices α1, α3, α4, α5 and in loops between helices α3 and α4, and between α5 and α6, form the binding cavity. Binding cavities in the OBPs homodimers form channels, a long and continuous hydrophobic tunnel, where the odorant binds [12]. We did not find any site under positive selection in *Anastrepha* that correspond to sites that form the binding cavity in *AaegOBP1*. The site under positive selection in *AfraOBP56h-1* and *AoblOBP56h-1* is located in α5 (Fig. 4a). The sites under positive selection in *AfraOBP56h-2* and *AoblOBP56h-2* (Fig. 4b) are located in α2, α3 e α6, in which the latter two helices bear sites that lead to changes in the physicochemical properties, according to analysis in PRIME.

The tertiary structure of *A. gambiae OBP4* (*AgamOBP4*) [63] was selected by Phyre2 as a reference for *AoblOBP57c* and *AfraOBP57c. AgamOBP4* is a protein with 124 amino acid residues, in which three disulphide bridges link α1 to α3, the start of α3 to α6, and the middle of α6 to α5. In *Anastrepha* (Fig. 4c), the positively selected sites were found in α2, α3, α4 and α6, and loops between α1 and α2, and between α3 and α4. Notably the positively selected amino acids located in α3 and especially in α6 may be structurally very important because they are located near disulphide bonds, specially the last one that causes changes in the chemical composition.

*A. gambiae* plus-C *OBP48*, selected by Phyre2 as reference for *Anastrepha OBP50a*, has 172 residues and eight α-helices that are stabilized by six disulphide bridges [14]. *AgamOBP48* is structurally divided into three domains: the “core”, the “NC-term” and the “cap”. The central “core” domain shares a common architecture with classic OBPs and consists of four bridged α-helices (α1, α3, α5 and α7), bearing six conserved cysteines that form three disulphide bridges connecting α1 to α3, α3 to the start of α7, and the middle of α7 to α5. This domain also contains two non-bridged α-helices α2 and α4, and a loop of eight amino acids (50s loop). Sites under positive selection in *Anastrepha* OBP50a sequences are found in the “core” region, in α3 and α7, in the loop between α2 and α3, and in the loop between α3 and β-sheet1 (Fig. 4d). A seventh cysteine in *AgamOBP48* is located at the top of α3, but it is not involved in a disulphide bond, since not all cysteine residues are necessarily involved in disulphide bonds [14]. For instance, *A. fraterculus* and *A. obliqua* plus-C *OBP50a* lost the twelfth conserved cysteine residue in a positively selected change that may have relevant structural consequences, but this loss does not seem to affect their function.

The “NC-term” domain comprises the N-terminal residues 1–25, the C-terminal 150 s loop (148–155) and the following C-terminal α8 helix (156–172), forming three disulphide bonds. The two subunits orient themselves so that the “NC-term” domain of one monomer inserts into the center of the neighboring monomer, to form a compact homodimer. The N-terminal component of this domain is characterized by two additional cysteines located in adjacent positions before C1 [14], pattern also observed in all *Anastrepha* plus-C OBPs. *Anastrepha OBP50a* has sites under positive selection in this domain, two ones in α8 and another one in the loop (Fig. 4d). Finally, the “Cap” domain, in which we failed to find sites under positive selection in *Anastrepha*, encompasses helix α6 and the loop near amino acid 120, and this interaction between the helix and the loop is characterized by multiple hydrogen-bonding that apparently serves to stabilize protein structure [14].

*AgamOBP48* shows a single binding site, although it is argued that given the symmetry of the dimer, ligand binding to two equivalent binding sites, named “NC-term” pockets, cannot be excluded [14]. We focused only on the single binding site to compare with *Anastrepha OBP50a*. The binding cavity is formed by 23 amino acid residues in the helices α5, α6 and α7, and in the loops between helices α2 and α3, and between α7 and α8. We found one amino acid under positive selection in the position exactly correspondent to the amino acid that forms the binding cavity in α7 of *AgamOBP48*. The other amino acid in α7, as well as the amino acids in the loop between α2 and α3, and in the loop between α7 and α8 did not correspond to the same position to the binding cavity, but they are juxtaposed to the amino acids that form it.

Because we are only using computational inferences, we cannot be certain about the 3D structures inferred for *Anastrepha OBP56h*, *OBP57c* and *OBP50a*, nor if the *Anastrepha* OBP binding cavity is formed in the same site positions that in the 3D structures of *Ae. aegypti* and *A. gambiae* OBPs used as reference. However, the OBPs’ high conservation at the structural level, observed in all insects studied, provide us some basis for this extrapolation [21, 22, 78]. The exception to this structure conservation is a unique feature of mosquito OBPs, their C-terminal loop covers the binding cavity, forming a “lid” for the release of ligands [79], therefore we expect that the C-terminal in *Anastrepha* may be quite different, potentially altering our 3D inference for the C-terminal part. Only *Anastrepha OBP50a* showed one amino acid under positive selection that possibly correspond to amino acids that form the binding cavity for odors. However, even if no replacement occurs in amino acids that are directly involved with the binding function, radical non-synonymous substitutions placed both in the α-helix or even in the loops are important because they might alter the size and shape of the binding cavity. For instance, by modifying the position of the disulphide bonds, as demonstrated elsewhere [21, 80].

Although the interaction mechanisms between OBPs and the OBP/ligand complex with ORs are still not well understood, studies showed that after binding the ligand, some OBPs are induced to a conformational change. These pH-dependent conformational changes were associated with changes in binding affinity, and it was reported to be common to some OBPs in distinct insects [12, 79, 81–84]. The OBP/ligand complex releases the ligand after they reach specific odorant receptors [75]. Therefore, amino acid changes that lead to conformational changes, such as those found in *A. fraterculus* and *A. obliqua* OBPs may also interfere with the interactions between OBPs and ORs, even when they are not directly involved with the binding sites.

### OBPs’ molecular evolution in *A. fraterculus* and *A. obliqua* may reflect specific adaptation

Despite the similar number of OBPs and their sequences, there is significant difference between OBPs of *A. obliqua* and *A. fraterculus* that may reflect specific adaptation. A wind tunnel test revealed that *A. fraterculus* antennae were responsive to Myrtaceae extracts, which also affected its oviposition rate [85]. On the other hand, adults of *A. obliqua* were attracted to Anacardiaceae ripe fruits [86, 87], so much so that at least nine volatile compounds from the Anacardiaceae *Spondias mombin* elicited antennal response from both sexes of this species [86]. Therefore, despite being considered generalists [88], both species have their preferences and show some host specificity for oviposition and feeding. Species of *Anastrepha* in general show lekking behavior [89], which has been described for *A. obliqua* [89, 90], as well as *A. fraterculus* [91]. In these leks, a number of males compete for space and display to have access to females. Several factors have been associated with success in the leks, chief among them their pheromones [91]. A comparison revealed that even though *A. fraterculus* and *A. obliqua* shared common chemical compounds on their pheromones, they also showed several different compounds emitted by calling males [87]. These ecological and reproductive differences may have been the driving force behind the rapid rates of evolution we identified amongst their OBP sequences, and suggest that the evolution of OBP genes may have had a significant impact in the evolution of species differences in this group.

## Conclusions

In this study, we used transcriptome data to identify over 23 different OBP genes in *A. fraterculus* and *A. obliqua*, which is the largest and the most diverse number of OBPs yet reported for a Tephritidae. We found great similarity in amino acid and DNA sequences among orthologous OBPs in *A. fraterculus* and *A. obliqua*, which may be a reflection of their recent divergence or evolutionary conservatism. However, OBPs from *A. fraterculus* and *A. obliqua* showed a faster rate of evolution when comparing to other genes among these species, a higher Ka/Ks ratio and evidence of positive selection on at least four *Anastrepha* OBP genes: *OBP56h-1*, *OBP56h-2, OBP57c* and *OBP50a*. We also found four positively selected sites in which site-specific changes would radically change amino acid properties, and likely promote structural and functional changes. One amino acid under positive selection in *OBP50a* is located in the binding cavity according the putative 3D-structure inference, which is important because such change may promote functional divergence of the binding specificities, and enable this protein to recognize and bind a new odorant. The other changes that are not directly involved with the binding function may also be important because they may alter the size and shape of the binding cavity or the solubility of the whole molecule. Considering that, as was shown in other insects, few amino acid changes in OBPs may result in significant differences in olfactory responses, our results stress out the importance of OBPs for the evolution and divergence of *A. fraterculus* and *A. obliqua*.

## Additional files

Additional file 1: Odorant-binding protein genes from *Ceratitis capitata*, *Drosophila melanogaster*, *Bactrocera dorsalis*, *Rhagoletis suavis* and *Rhagoletis pomonella* used in the phylogenetic analyses. (XLSX 10 kb)
Additional file 2: Non-rooted phylogenetic trees by subfamily used in PAML evolutionary analysis. Each colored branch represents a different branch-site test analysis. Branch lengths are estimated by amino acid substitutions per site. (JPG 241 kb)

## References

[1] Orr HA, Irving S (2001). Complex epistasis and the genetic basis of hybrid sterility in the *Drosophila pseudoobscura* Bogota-USA hybridization. Genetics.

[2] Noor MAF, Feder JL (2006). Speciation genetics: evolving approaches. Nat Rev Genet.

[3] Rice WR (1992). Sexually antagonistic genes: experimental evidence. Science.

[4] Swanson WJ, Vacquier VD (2002). The rapid evolution of reproductive proteins. Nat Rev Genet.

[5] Forêt S, Maleszka R (2006). Function and evolution of a gene family encoding odorant binding-like proteins in a social insect, the honey bee (*Apis mellifera*). Genome Res.

[6] Gardiner A, Barker D, Butlin RK, Jordan WC, Ritchie MG (2008). *Drosophila* chemoreceptor gene evolution: selection, specialization and genome size. Mol Ecol.

[7] Kosiol C, Vinař T, Fonseca RR, Hubisz MJ, Bustamante CD, Nielsen R (2008). Patterns of positive selection in six mammalian genomes. PLoS Genet.

[8] Sánchez-Gracia A, Rozas J (2008). Divergent evolution and molecular adaptation in the *Drosophila* odorant-binding protein family: inferences from sequence variation at the OS-E and OS-F genes. BMC Evol Biol.

[9] Benton R (2006). On the ORigin of smell: odorant receptors in insects. Cell Mol Life Sci.

[10] Vogt RG, Riddiford LM (1981). Pheromone binding and inactivation by moth antennae. Nature.

[11] Pelosi P, Maida R (1995). Odorant-binding proteins in insects. Comp Biochem Physiol B Biochem Mol Biol.

[12] Leite NR, Krogh R, Xu W, Ishida Y, Iulek J, Leal WS (2009). Structure of an odorant-binding protein from the mosquito *Aedes aegypti* suggests a binding pocket covered by a pH-sensitive ‘lid’. PLoS One.

[13] Lagarde A, Spinelli S, Tegoni M, He XL, Field L, Zhou J-J (2011). The crystal structure of odorant binding protein 7 from *Anopheles gambiae* exhibits an outstanding adaptability of its binding site. J Mol Biol.

[14] Tsitsanou KE, Drakou CE, Thireou T, Gruber AV, Kythreoti G, Azem A (2013). Crystal and solution studies of the ‘Plus-C’ odorant-binding protein 48 from *Anopheles gambiae*: control of binding specificity through three-dimensional domain swapping. J Biol Chem.

[15] Hekmat-Scafe DS, Scafe CR, McKinney AJ, Tanouye MA (2002). Genome-wide analysis of the odorant-binding protein gene family in *Drosophila melanogaster*. Genome Res.

[16] Zhou J-J, Zhang GA, Huang W, Birkett MA, Field LM, Pickett JA (2004). Revisiting the odorant-binding protein LUSH of *Drosophila melanogaster*: evidence for odour recognition and discrimination. FEBS Lett.

[17] Vieira FG, Forêt S, He XL, Rozas J, Field LM (2012). Unique features of odorant-binding proteins of the parasitoid wasp *Nasonia vitripennis* revealed by genome annotation and comparative analyses. PLoS One.

[18] Valenzuela JG, Charlab R, Gonzalez EC, Miranda-Santos IKF, Marinotti O, Francischetti IMB (2002). The D7 family of salivary proteins in blood sucking Diptera. Insect Mol Biol.

[19] Vieira FG, Sánchez-Gracia A, Rozas J (2007). Comparative genomic analysis of the odorant-binding protein family in 12 *Drosophila* genomes: purifying selection and birth-and-death evolution. Genome Biol.

[20] Ohta S, Seto Y, Tamura K, Ishikawa Y, Matsuo T (2014). Identification of odorant-binding protein genes expressed in the antennae and the legs of the onion fly, *Delia antiqua* (Diptera: Anthomyiidae). Appl Entomol Zool.

[21] Tegoni M, Campanacci V, Cambillau C (2004). Structural aspects of sexual attraction and chemical communication in insects. Trends Biochem Sci.

[22] Galindo K, Smith DP (2001). A large family of divergent *Drosophila* odorant-binding proteins expressed in gustatory and olfactory sensilla. Genetics.

[23] Nei M, Rooney AP (2005). Concerted and birth-and-death evolution of multigene families. Annu Rev Genet.

[24] Sánchez-Gracia A, Aguadé M, Rozas J (2003). Patterns of nucleotide polymorphism and divergence in the odorant-binding protein genes OS-E and OS-F: analysis in the melanogaster species subgroup of *Drosophila*. Genetics.

[25] Perre P, Jorge LR, Lewinsohn TM, Zucchi RA (2014). Morphometric differentiation of fruit fly pest species of the *Anastrepha fraterculus* group (Diptera: Tephritidae). Ann Entomol Soc Am.

[26] Selivon D, Perondini ALP, Morgante JS (2005). A genetic–morphological characterization of two cryptic species of the *Anastrepha fraterculus* complex (Diptera: Tephritidae). Ann Entomol Soc Am.

[27] Pereira-Rêgo DRG, Jahnke SM, Redaelli LR, Schaffer N (2011). Morfometria de *Anastrepha fraterculus* (Wied.) (Diptera: Tephritidae) relacionada a hospedeiros nativos, Myrtaceae. Arq Inst Biol.

[28] Camargo CA, Odell E, Jirón LF (1996). Interspecific interactions and host preference of *Anastrepha obliqua* and *Ceratitis capitata* (Diptera: Tephritidae), two pests of mango in Central America. Fla Entomol.

[29] Matsuo T, Sugaya S, Yasukawa J, Aigaki T, Fuyama Y (2007). Odorant-binding proteins OBP57d and OBP57e affect taste perception and host-plant preference in *Drosophila sechellia*. PLoS Biol.

[30] Laughlin JD, Ha TS, Jones DNM, Smith DP (2008). Activation of pheromone-sensitive neurons is mediated by conformational activation of pheromone-binding protein. Cell.

[31] Rezende VB, Congrains CC, Lima ALA, Campanini EB, Nakamura AM, Oliveira JL (2016). Head transcriptomes of two closely related species of fruit flies of the *Anastrepha fraterculus* group reveals divergent genes in species with extensive gene flow. G3 Genes Genom Genet.

[32] Chomczynski P, Mackey K (1995). Short technical reports. Modification of the TRI reagent procedure for isolation of RNA from polysaccharide- and proteoglycan-rich sources. BioTechniques.

[33] SeqyClean. https://github.com/ibest/seqyclean. Accessed 14 Jan 2014

[34] Grabherr MG, Haas BJ, Yassour M, Levin JZ, Thompson DA, Amit I (2011). Full-Length transcriptome assembly from RNA-Seq data without a reference genome. Nat Biotechnol.

[35] Altschul SF, Gish W, Miller W, Myers EW, Lipman DJ (1990). Basic local alignment search tool. J Mol Evol.

[36] ORF finder. https://www.ncbi.nlm.nih.gov/orffinder/. Accessed 10 Feb 2014.

[37] Hiller K, Grote A, Scheer M, Münch R, Jahn D (2004). PrediSi: prediction of signal peptides and their cleavage positions. Nucleic Acid Res.

[38] Katoh K, Standley DM (2013). MAFFT multiple sequence alignment software version 7: improvements in performance and usability. Mol Biol Evol.

[39] Hall TA (1999). BioEdit: A user-friendly biological sequence alignment editor and analysis program for Windows 95/98/NT. Nucleic Acids Symp Ser.

[40] Smith JM (1992). Analyzing the mosaic structure of genes. J Mol Evol.

[41] Sawyer S (1989). Statistical tests for detecting gene conversion. Mol Biol Evol.

[42] Martin D, Rybicki E (2000). RDP: detection of recombination amongst aligned sequences. Bioinformatics.

[43] Martin DP, Lemey P, Lott M, Moulton V, Posada D, Lefeuvre P (2010). RDP3: a flexible and fast computer program for analyzing recombination. Bioinformatics.

[44] Posada D (2008). jModelTest: phylogenetic model averaging. Mol Biol Evol.

[45] Guindon S, Dufayard JF, Lefort V, Anisimova M, Hordijk W, Gascuel O (2010). New algorithms and methods to estimate maximum-likelihood phylogenies: assessing the performance of PhyML 3.0. Syst Biol.

[46] Zheng W, Peng W, Zhu C, Zhang Q, Saccone G, Zhang H (2013). Identification and expression profile analysis of odorant binding proteins in the oriental fruit fly *Bactrocera dorsalis*. Int J Mol Sci.

[47] Schwarz D, Robertson HM, Feder JL, Varala K, Hudson ME, Ragland GJ (2009). Sympatric ecological speciation meets pyrosequencing: sampling the transcriptome of the apple maggot *Rhagoletis pomonella*. BMC Genomics.

[48] Ramsdell KMM, Lyons-Sobaski SA, Robertson HM, Walden KKO, Feder JL, Wanner K (2010). Expressed sequence tags from cephalic chemosensory organs of the northern walnut husk fly, *Rhagoletis suavis*, including a putative canonical odorant receptor. J Insect Sci.

[49] Larkin MA, Blackshields G, Brown NP, Chenna R, McGettigan PA, McWilliam H (2007). Clustal W and Clustal X version 2.0. Bioinformatics.

[50] Zhou J-J, He XL, Pickett JA, Field LM (2008). Identification of odorant-binding proteins of the yellow fever mosquito *Aedes aegypti*: genome annotation and comparative analyses. Insect Mol Biol.

[51] Gong DP, Zhang HJ, Zhao P, Xia QY, Xiang ZH (2009). The odorant binding protein gene family from the genome of silkworm, *Bombyx mori*. BMC Genomics.

[52] Zhou J-J, Vieira FG, He XL, Smadja C, Liu R, Rozas J (2010). Genome annotation and comparative analyses of the odorant-binding proteins and chemosensory proteins in the pea aphid *Acyrthosiphon pisum*. Insect Mol Biol.

[53] Xia X (2013). DAMBE5: a comprehensive software package for data analysis in molecular biology and evolution. Mol Biol Evol.

[54] Zhang J (2005). Evaluation of an improved branch-site likelihood method for detecting positive selection at the molecular level. Mol Biol Evol.

[55] Yang Z (2007). PAML 4: phylogenetic analysis by maximum likelihood. Mol Biol Evol.

[56] Anisimova M, Bielawsky JP, Yang Z (2001). Accuracy and power of the likelihood ratio test in detecting adaptive molecular evolution. Mol Biol Evol.

[57] Zhang Z, Li J, Zhao X-Q, Wang J, Wong GK-S, Yu J (2006). KaKs_Calculator: calculating Ka and Ks through model selection and model averaging. Genomics Proteomics Bioinformatics.

[58] Posada D, Baxevanis AD, Davison DB, Page RDM, Petsko GA, Stein LD, Stormo GD (2003). Using MODELTEST and PAUP* to select a model of nucleotide substitution. Current protocols in bioinformatics.

[59] Swanson WJ, Wong A, Wolfner MF, Aquadro CF (2004). Evolutionary expressed sequence tag analysis of *Drosophila* female reproductive tracts identifies genes subjected to positive selection. Genetics.

[60] Conant GC, Wagner GP, Stadler PF (2007). Modeling amino acid substitution patterns in orthologous and paralogous genes. Mol Phylogenet Evol.

[61] Delport W, Poon AF, Frost SDW, Kosakovsky-Pond SL (2010). Datamonkey 2010: a suite of phylogenetic analysis tools for evolutionary biology. Bioinformatics.

[62] Kelley LA, Sternberg MJE (2009). Protein structure prediction on the web: a case study using the phyre server. Nat Protoc.

[63] Davrazou F, Dong E, Murphy EJ, Jones DNM. Conformational ordering plays a key role in regulating heterodimeric interactions between odorant binding proteins from *Anopheles gambiae*. http://www.rcsb.org/pdb/explore.do?structureId=3q8i. Accessed 17 Sep 2014

[64] Zhu J-Y, Zhang L-F, Ze S-Z, Wang D-W, Yang B (2013). Identification and tissue distribution of odorant binding protein genes in the beet armyworm, *Spodoptera exigua*. J Insect Physiol.

[65] Manoharan M, Chong MNF, Vaïtinadapoulé A, Frumence E, Sowdhamini R, Offmann B (2013). Comparative genomics of odorant binding proteins in *Anopheles gambiae*, *Aedes aegypti*, and *Culex quinquefasciatus*. Genome Biol Evol.

[66] Kirkness EF, Haas BJ, Sun W, Braig HR, Perotti MA, Clark JM (2010). Genome sequences of the human body louse and its primary endosymbiont provide insights into the permanent parasitic lifestyle. Proc Natl Acad Sci U S A.

[67] Vieira FG, Rozas J (2011). Comparative genomics of the odorant-binding and chemosensory protein gene families across the Arthropoda: origin and evolutionary history of the chemosensory system. Genome Biol Evol.

[68] Qiao H, Tuccori E, He XL, Gazzano A, Field L, Zhou JJ (2009). Discrimination of alarm pheromone (E)-beta-farnesene by *Aphid* odorant-binding proteins. Insect Biochem Molec.

[69] Wang S-Y, Gu S-H, Han L, Guo Y-Y, Zhou J-J, Zhang Y-J (2013). Specific involvement of two amino acid residues in cis-nerolidol binding to odorant-binding protein 5 AlinOBP5 in the alfalfa plant bug, *Adelphocoris lineolatus* (Goeze). Insect Mol Biol.

[70] Ahmed T, Zhang T-T, Wang Z-Y, He K-L, Bai S-X (2014). Three amino acid residues bind corn odorants to McinOBP1 in the polyembryonic endoparasitoid of *Macrocentrus cingulum* brischke. PLoS One.

[71] Wang P, Lyman RF, Shabalina SA, Mackay TFC, Anholt RRH (2007). Association of polymorphisms in odorant-binding protein genes with variation in olfactory response to benzaldehyde in *Drosophila*. Genetics.

[72] Anisimova M, Nielsen R, Yang Z (2003). Effect of recombination on the accuracy of the likelihood method for detecting positive selection at amino acid sites. Genetics.

[73] Gotzek D, Robertson HM, Wurm Y, Shoemaker D (2011). Odorant binding proteins of the red imported fire ant, *Solenopsis invicta*: an example of the problems facing the analysis of widely divergent proteins. PLoS One.

[74] Siciliano P, Scolari F, Gomulski LM, Falchetto M, Manni M, Gabrieli P, Field LM, Zhou J-J, Gasperi G, Malacrida AR (2014). Sniffing out chemosensory genes from the Mediterranean fruit fly, *Ceratitis capitata*. PLoS One.

[75] Fan J, Francis F, Liu Y, Chen JL, Cheng DF (2011). An overview of odorant-binding protein functions in insect peripheral olfactory reception. Genet Mol Res.

[76] Fletcher W, Yang Z (2010). The effect of insertions, deletions, and alignment errors on the branch-site test of positive selection. Mol Biol Evol.

[77] Kryazhimskiy S, Plotkin JB (2008). The population genetics of dN/dS. Edited by Takashi Gojobori. PLoS Genet.

[78] Zhou J-J, Robertson G, He X, Dufour S, Hooper AM, Pickett JA, Keep NH, Field LM (2009). Characterisation of *Bombyx mori* odorant-binding proteins reveals that a general odorant-binding protein discriminates between sex pheromone components. J Mol Biol.

[79] Wogulis M, Morgan T, Ishida Y, Leal WS, Wilson DK (2006). The crystal structure of an odorant binding protein from *Anopheles gambiae*: evidence for a common ligand release mechanism. Biochem Bioph Res Co.

[80] Lartigue A (2003). The crystal structure of a cockroach pheromone-binding protein suggests a new ligand binding and release mechanism. J Biol Chem.

[81] Sandler BH, Nikonova L, Leal WS, Clardy J (2000). Sexual attraction in the silkworm moth: structure of the pheromone-binding-protein-bombykol complex. Chem Biol.

[82] Xu W, Leal WS (2008). Molecular switches for pheromone release from a moth pheromone-binding protein. Biochem Biophys Res Commun.

[83] Mao Y, Xu X, Xu W, Ishida Y, Leal WS, Ames JB, Clardy J (2010). Crystal and solution structures of an Odorant-binding protein from the southern house mosquito complexed with an oviposition pheromone. Proc Natl Acad Sci U S A.

[84] Xu X, Xu W, Rayo J, Ishida Y, Leal WS, Ames JB (2010). NMR structure of navel orangeworm moth pheromone-binding protein (AtraPBP1): implications for pH-sensitive pheromone detection. Biochemistry.

[85] Gregorio PLF, Sant’Ana J, Redaelli LR (2010). Percepção química e visual de *Anastrepha fraterculus* (Diptera, Tephritidae) em laboratório. Iheringia Sér Zool.

[86] Cruz-López L, Malo EA, Toledo J, Virgen A, Mazo A, Rojasm JC (2006). A new potential attractant for *Anastrepha obliqua* from *Spondias mombin* fruits. J Chem Ecol.

[87] López-Guillén G, López LC, Malo EA, Rojas JC (2011). Olfactory responses of *Anastrepha obliqua* (Diptera: Tephritidae) to volatiles emitted by calling males. Fla Entomol.

[88] Morgante JS, Selivon D, Solferini VN, Matioli SR, Aluja M, Liedo P (1993). Evolutionary patterns in specialist and generalist species of *Anastrepha*. Fruit flies: biology and management.

[89] Aluja M, Piñeiro J, Jácome I, Díaz-Fleischer F, Sivinski J, Aluja M, Norrbom AL (2000). Behavior of flies in the genus *Anastrepha* (Trypetinae: Toxotripanini). Fruit flies (Tephritidae): phylogeny and evolution of behavior.

[90] Aluja M, Cabrera M, Guillen J, Celedonio H, Ayora F (1989). Behaviour of *Anastrepha ludens*, *A. obliqua* and *A. serpentina* (Diptera:Tephritidae) on a wild mango tree (*Mangifera indica*) harbouring three McPhail traps. Int J Trop Insect Sci.

[91] Segura DF, Petit-Marty N, Sciurano R, Vera T, Calcagno G, Allinghi A, Cendra PG, Cladera JL, Vilardi J (2007). Lekking behavior of *Anastrepha fraterculus* (Diptera: Tephritidae). Fla Entomol.

