# Supplementary figures and images for: Molecular evolution of Odorant-binding proteins gene family in two closely related Anastrepha fruit flies
Source: BMC Evol Biol. 2016 Oct 7;16:198. doi: 10.1186/s12862-016-0775-0 (PMC5054612; doi:10.1186/s12862-016-0775-0)

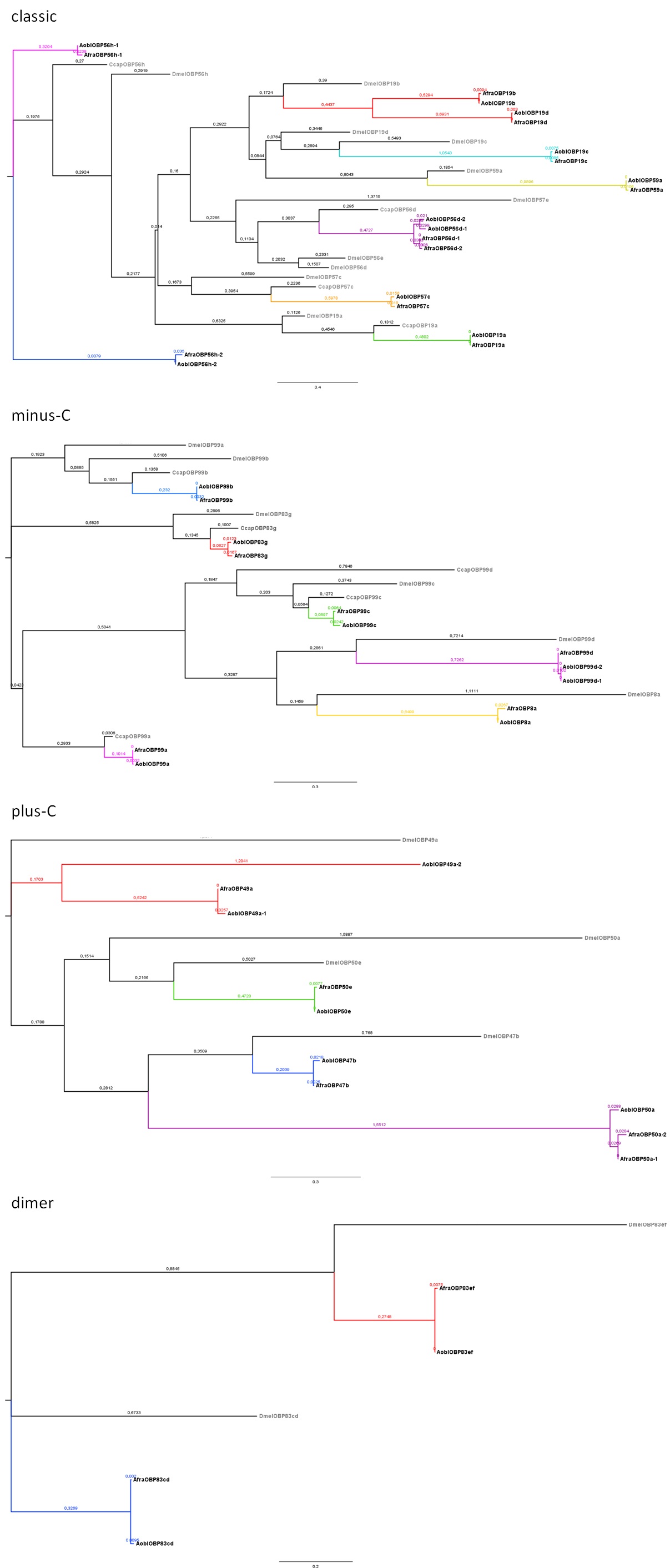

Supplement: Additional file 2: — Non-rooted phylogenetic trees by subfamily used in PAML evolutionary analysis. Each colored branch represents a different branch-site test analysis. Branch lengths are estimated by amino acid substitutions per site. (JPG 241 kb) [file 12862_2016_775_MOESM2_ESM.jpg]
